# Three-Dimensional Digital Light-Processing Bioprinting Using Silk Fibroin-Based Bio-Ink: Recent Advancements in Biomedical Applications

**DOI:** 10.3390/biomedicines10123224

**Published:** 2022-12-12

**Authors:** Md. Tipu Sultan, Ok Joo Lee, Joong Seob Lee, Chan Hum Park

**Affiliations:** 1 Nano-Bio Regenerative Medical Institute (NBRM), College of Medicine, Hallym University, Chuncheon 24252, Republic of Korea; 2 Department of Otorhinolaryngology-Head & Neck Surgery, Hallym University Sacred Heart Hospital, Anyang 14068, Republic of Korea; 3 Depratment of Otorhinolaryngology-Head and Neck Surgery, Chuncheon Sacred Heart Hospital, Chuncheon 24253, Republic of Korea

**Keywords:** three-dimensional bioprinting, digital light processing, silk fibroin, bio-ink, biomedical application

## Abstract

Three-dimensional (3D) bioprinting has been developed as a viable method for fabricating functional tissues and organs by precisely spatially arranging biomaterials, cells, and biochemical components in a layer-by-layer fashion. Among the various bioprinting strategies, digital light-processing (DLP) printing has gained enormous attention due to its applications in tissue engineering and biomedical fields. It allows for high spatial resolution and the rapid printing of complex structures. Although bio-ink is a critical aspect of 3D bioprinting, only a few bio-inks have been used for DLP bioprinting in contrast to the number of bio-inks employed for other bioprinters. Recently, silk fibroin (SF), as a natural bio-ink material used for DLP 3D bioprinting, has gained extensive attention with respect to biomedical applications due to its biocompatibility and mechanical properties. This review introduces DLP-based 3D bioprinting, its related technology, and the fabrication process of silk fibroin-based bio-ink. Then, we summarize the applications of DLP 3D bioprinting based on SF-based bio-ink in the tissue engineering and biomedical fields. We also discuss the current limitations and future perspectives of DLP 3D bioprinting using SF-based bio-ink.

## 1. Introduction

Over the last few decades, 3D-printing technology has quickly advanced in many fields, including aerospace, product manufacturing, art, and other industries [1]. Three-dimensional printing was first presented in 1986 and is regarded as an additive manufacturing process [2]. Through the employment of digital files, 3D-printing technology has merits in manufacturing complex structures by depositing materials such as ceramic, metal, plastics, and natural and synthetic polymers [3]. These files are commonly produced by computers via 3D-modeling software, e.g., computer-aided design (CAD). The general 3D-printing process includes CAD file production, conversion to an STL file, transferring this file to a 3D printer, and then processing it via 3D printing [4,5].

Bioprinting is gaining popularity as a means of customizing the structures of scaffolds and managing cells, the delivery of bioactive substances, and the regeneration of tissue. The most significant aspects of the application of this technology are its printing equipment/methods and materials. The commonly used methods for 3D printing include extrusion, inkjet, and light-assisted 3D printing [6,7,8]. Extrusion methods push a printing material to a nozzle using pneumatic or mechanical force (a screwdriver or piston). Inkjet 3D printing incorporates droplets from a thermal actuator to construct 3D objects. Both inkjet and extrusion methods have merits in their simplicity, flexibility, and low cost [9]. However, these two printing methods have several limitations (Table 1.). Due to shear stress and the small orifices of nozzles, cell damage and aggregation frequently occur in the 3D-bioprinting field. In addition, printing resolution is limited due to the confinement of the physical nozzle size (generally > 50 μm) [10].

DLP printing, also known as digital micro-mirror device (DMD)-based stereolithography, which was invented by Lu et al., has been expanded as a high-throughput system that provides high spatial resolution with excellent biocompatibility [13]. The DLP 3D-printing method employs light to polymerize materials in order to obtain 3D structures. In this 3D-printing method, ultraviolet (UV) light is commonly used to photopolymerize the precursor solution of the polymer and produce a 3D scaffold in a layer-by-layer system [14]. Contrary to extrusion and inkjet-based 3D-printing methods, DLP 3D printing has substantial benefits with respect to printing fidelity and processing time. The disadvantages of DLP printing are its relatively high printing cost and the limited availability of the printing materials used in the process (only photopolymerizable materials) [15,16].

Currently, the selection of biomaterials for use as a bio-ink constitutes another critical issue in 3D printing. Therefore, many researchers have sought for suitable materials to use as bio-ink in 3D printing. Adequate mechanical properties and structural integrity are the prerequisites for an ideal 3D-printing material. In addition, if the 3D product is intended for use in the biomedical field, it should have excellent biocompatibility [17]. Many synthetic polymers are frequently employed as bio-inks in 3D printing, and they have the advantages of convenient synthesis, easy acquisition, easy processing, low cost, and sufficient stress tolerance [18]. However, they also have the disadvantages of poor biocompatibility, poor cellular adhesion, and the production of toxic by-products during degradation [19]. Natural biomaterials have gained a great deal of attention owing to the drawbacks of synthetic polymers. Among the popular biological macromolecules used to create bio-inks for 3D printing are gelatin, alginate, fibrinogen, fibrin, collagen, chitosan, and their mixtures [20,21,22,23,24,25,26]. Although natural biomaterials tend to have superb biocompatibility, they also have shortcomings, namely, poor printability and mechanical strength [27,28] (Table 2).

**Figure 1:**
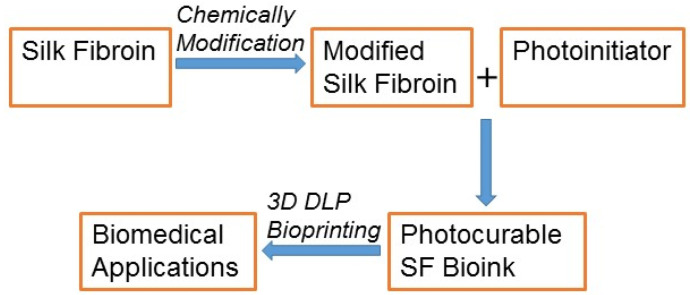
Schematic overview of the biomedical application of the SF-based bio-ink via 3D DLP printing.

Silk fibroin (SF) is a natural polymer produced from the silkworm *Bombyx mori*, which was approved for use as a biomaterial in 1993 by the FDA. Recently, it has been extensively applied in the tissue engineering and biomedical fields owing to its exceptional biocompatibility, flexible mechanical properties, controlled biodegradation, and minimal propensity to invoke immune reactions [32,33,34]. SF comprises a repetition of Gly–Ala–Gly–Ala–Gly–Ser repeats of amino acid sequences, forming a β-sheet structure by self-assembly [35,36]. For numerous biomedical applications, SF has been fashioned into several forms such as sponges, films, hydrogels, electrospun mats, and nano- or microparticles [37,38]. Owing to its versatility, SF has been employed as a significant bio-ink material for various 3D-printing approaches, including inkjet and extrusion-based bioprinting [39,40]. Nevertheless, SF’s low viscosity and the low abundance of SF are significant drawbacks of its use as bio-ink for general 3D-printing applications. Hence, SF is being implemented as a bio-ink for 3D bioprinting by mixing it with other high-viscosity materials to enhance its printability [15,30,41]. The use of additional crosslinking to natural biomaterials is a common strategy with which to improve printability and mechanical properties. Photo-crosslinking, a cross-linking method, has provided fast and robust curing by creating additional intra- and inter-molecular chemical bonds [42]. Recently, we developed several SF-based bio-inks chemically modified by glycidyl methacrylate for DLP 3D printing that have been vastly employed in the biomedical field owing to their remarkable biochemical and biological properties for several biomedical uses [43]. The developmental process of the various SF-based bio-inks for 3D DLP printers and their use in several biomedical fields has been demonstrated in detail in our recently published protocol [44]. This review emphases the recent DLP 3D-printable SF-based bio-ink and its applications in contemporary biomedical research (Figure 1).

## 2. DLP 3D-Printing Technology and Development of Silk-Based Bio-Ink

### 2.1. DLP 3D-Printing Technology

**Figure 2:**
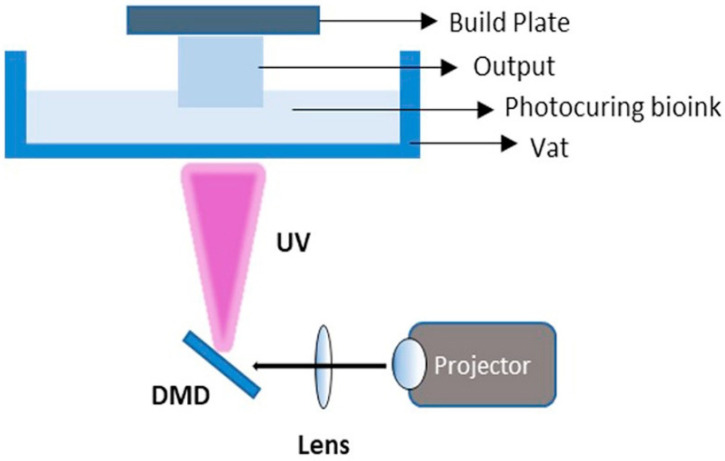
Schematic illustration of DLP-printing process. DMD: Digital micromirror device. Adapted from [39] with permission from Elsevier.

In 1984, the birth of 3D printing took place when Charles Hull used stereolithography (SLA) to print 3D objects from digital data. In 1987, direct light processing was invented. In 1988, Klebe first revealed bioprinting by deposition through cyto-scribing technology using a Hewlett-Packard inkjet printer [45]. To create complex anatomy by depositing cells, Odde and Renn first applied laser-assisted bioprinting in 1999 [46]. DLP 3D-printing technology, invented by Texas instruments, originated from image projection technology [10]. The digital micro-mirror device (DMD) is the main functional segment of the general DLP printer. The DMD is a complex of micron-sized, well-regulated mirrors (Figure 2) [47]. The mirrors can be independently revolved ±12° to an on or off-state to manage the path of the light. Then, reflected light is projected into the photosensitive bio-ink during operation. The resolution of DLP 3D printing is usually between 15 and 100 μm and is related to the quality and pixel resolution of the DMD and the accuracy of the construction plate [48].

This printing system works in a top-down or bottom-up layout. DLP technology has several advantages over the previously mentioned methods. First, projection technology permits layer-by-layer polymerization. Consequently, the construction time is quicker (~30 min; mm^3^·s^−1^) than other printing methods, including extrusion, inject, and laser micro-stereolithography with line-by-line forms (). Regardless of the complexity or size, the printing time of each layer is the same and only depends on the depth of the construction. These relatively fast printing times can result in excellent cell viability (85–95%). Since its printing process does not include exposure to high temperatures, pressure, or shear stress, this DLP printing technology provides suitable circumstances for cells in 3D bioprinting.

### 2.2. Development of Photo-Cross-Linkable SF-Based Biomaterial for DLP 3D Printing

DLP 3D printers have the advantages of a fast printing time and high resolution and are biocompatible with nozzle-free printing. However, the use of this technology is quite limited due to the inadequate light-sensitive biomaterials it incorporates. Photopolymerized biomaterials must be used in the printing process, and the number of functional groups for photopolymerization found in natural biomaterials is quite inadequate. Several natural and synthetic polymer-based bio-inks, including gelatin, hyaluronic acid (HA), polyvinyl alcohol, and polyethylene glycol, have been used for DLP 3D printing.

Gelatin is a natural polymer found in the skin, cartilage, and tendons of animals. It is a molecular collagen derivative produced by collagen’s unalterable denaturation. The molecular structure and function of gelatin are very similar to collagen. Therefore, it is frequently employed in tissue engineering for biomaterial purposes [49]. Gelatin has several advantages, including inherent Arg-Gly-Asp (RGD) motifs, low immunogenicity, biocompatibility, and biodegradability. However, due to its inadequate mechanical properties, it is very difficult to employ gelatin for printing purposes. Gelatin is extremely temperature-sensitive. Since it can be dissolved in water above 40 °C and below this temperature it can be transformed into a gel, a special control system is required during the printing process [50]. In the 2000s, methacrylated gelatin (GelMA) was invented to solve this problem. GelMA has been conjugated with methyl methacrylate (MA) for photocrosslinking. MA or glycidyl methacrylate (GMA) is commonly utilized to produce C=C double bonds in biomaterials [51]. Following the development of GelMA, other methacrylated biomaterials have been reported, including pectin methacrylate, methacrylated hyaluronic acid, and glycidyl-methacrylated SF (SGMA). After the addition of a photo-initiator, these biomaterials can be photo-crosslinked using UV light. A photo-initiator is a critical component that absorbs the energy of light at a specified frequency and produces radicals that transform the monomer solution into polymers [52]. Igracure-2959, lithium phenyl-2,4,6-trimethylbenzoylphosphinate (LAP), and eosin Y are the three of the most frequent photo-initiators used in bioprinting [53]. In DLP printing, the selection of a photo-initiator is crucial because it influences the efficiency of polymerization, which affects the printing time, power, and resolution [54].

HA, a type of polysaccharide, has been extensively applied in wound healing, angiogenesis, tendon regeneration, and cartilage regeneration [55,56,57,58,59]. It is less immunogenic. It can be formed in hydrogels through various crosslinked mechanisms. Generally, it is applied in combination with another material or is chemically altered due to its weakness in water [31]. Methacrylated HA has shown a rapid degradation period of about only 2 days [55]. In addition, HA has been demonstrated to be more appropriate for extrusion-based bioprinters than DLP 3D bioprinters [60]. Therefore, the challenge remains to develop a suitable bio-ink for the DLP 3D bioprinter that possesses mechanically stable properties and is biocompatible, biodegradable, and bio-printable.

After the FDA approved SF as a biomaterial in 1993, it has been extensively applied as a scaffold in biomedical fields due to its excellent biocompatibility, notable mechanical properties, and adjustable biodegradability [61,62]. SF can be integrated with different materials via several chemical adjustments, such as amino acid, coupling, and grafting reactions. The addition of photopolymerizable methacryloyl groups into SF via amine-containing side groups allows for covalent crosslinking using UV exposure after the DLP printing procedure. Using methacrylation, Kim et al. recently developed an SF-based bio-ink for a DLP-bioprinting procedure [43].

**Figure 3:**
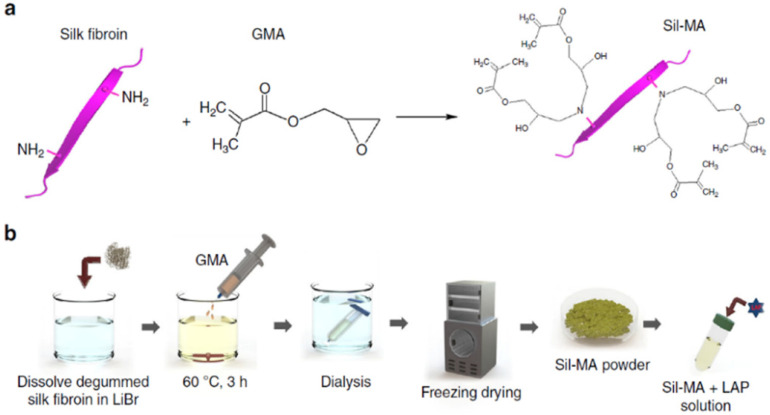
The fabrication process of SF bio-ink for DLP printing. (**a**) SF molecules were modified with the addition of GMA by methacrylation. (**b**) Graphical illustration of methacrylation of SF. GMA was added dropwise into the dissolved SF solution in LiBr (9.5 M) and mixed with continuous stirring at 60 °C for 3 h. Then, it was dialyzed against distilled water for 4 days and freeze-dried. Finally, LAP was put into a GMA-modified SF solution. Adapted from [43] with permission from Springer Nature.

SF solution was obtained by degumming the sliced silk cocoons (40 g) in 1 L of sodium carbonate (Na2CO3) solution (0.05 M) by boiling them at 100 °C for 30 min. A total of 20 g of room-temperature-dried, degummed SF was dissolved at 60 °C for 1 h in 100 mL solution of (9.3 M) lithium bromide (LiBr) (Figure 3). Then, glycidyl methacrylate (GMA) solution (141–705 mM) was slowly poured into this dissolved SF in a LiBr mixture with continuous stirring (300 rpm) for 3 h at 60 °C to produce a reaction between SF and GMA. After that, the resultant solution was dialyzed against distilled water using cutoff dialysis tubes of 12–14 kDa for 7 days. Then, the resultant solutions of methacrylated SF (Sil-MA) were dried using a freezer for 48 h. The degree of methacrylation was determined using NMR and FT-IR. Although methacrylic anhydride (MA) was used to modify gelatin [63] and HA [64] to insert methacryloyl groups, GMA is a more suitable reagent for SF modification than MA since GMA does not produce any acid as a by-product, which may crystalize unwanted steps in the SF modification process via the epoxide ring-opening process.

An Sil-MA bio-ink was prepared by adding 0.2 %w/v LAP to the 10–30% Sil-MA solution. Then, it was printed with a high-resolution DLP printer. LAP, started at 365 nm UV light, has improved water solubility with less cytotoxicity compared to Irgacure 2959. The visible light absorbance of LAP (at 400 nm) can polymerize using visible light. Using visible light as a light source, Applegate et al. polymerized SF using riboflavin (vitamin B2) as a photo-initiator via tyrosine in the SF protein [65]. Visible light is nontoxic to cells. However, its high infiltration ratio and extended cross-linking period disturb the ultimate configuration of the printed structure and are not favored by DLP printers.

**Figure 4:**
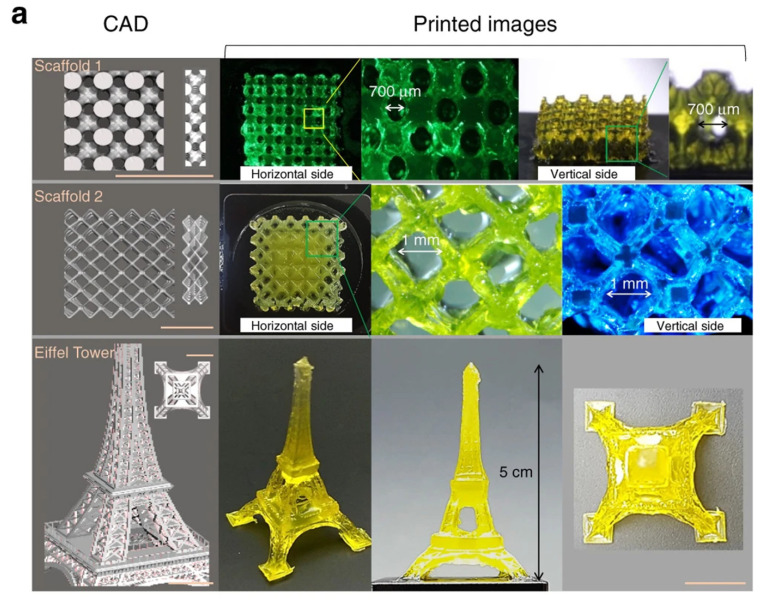

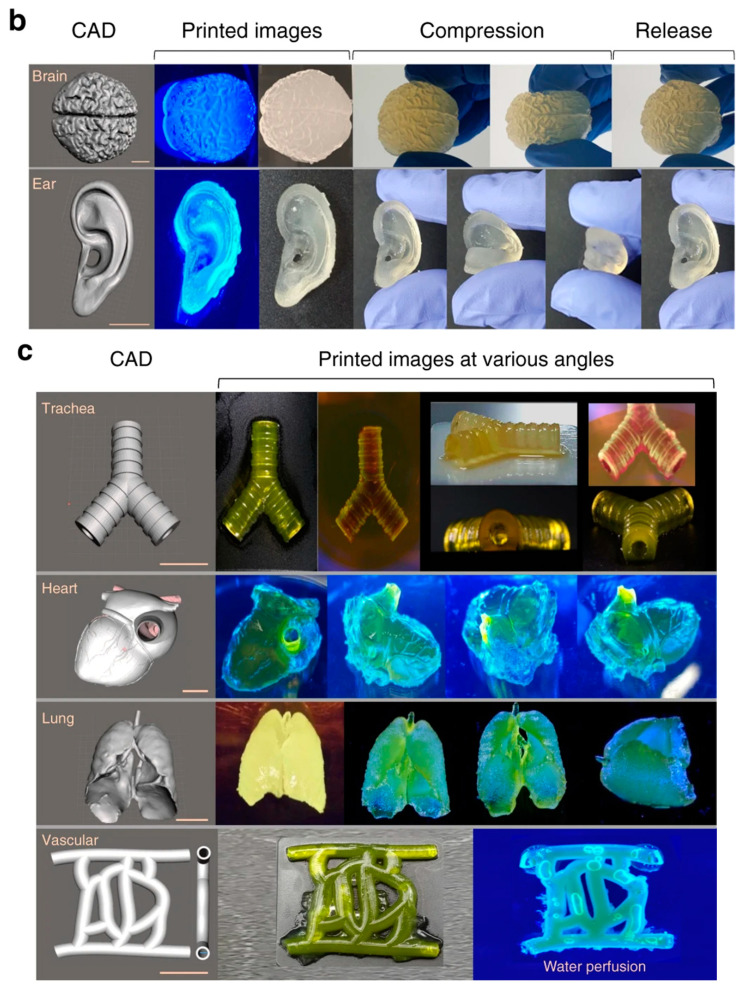
Sil-MA bio-ink’s printability via DLP bioprinter. (**a**) The simulated scaffold and the Eiffel Tower; (l) images of the CAD portraying the scaffolds and Eiffel Tower and (r) printed pictures. (**b**) Human ear and brain mirrored structures; (l) the images of the CAD showing the human ear and brain and (r) printed pictures. Printed constructs were not broken when firmly compacted using fingers, and when fingers were relaxed, they reverted to their primary structures. (**c**) The trachea, heart, lung, and blood vessels imitated morphology; (l) the CAD pictures representing human trachea, heart, lung, and blood vessels and (r) printed pictures at several angles. Printed yields using Sil-MA by DLP displayed complex constructions imitating their CAD pictures, e.g., arteries and veins. Scale bar indicates 1. Adapted from [43] with authorization from Springer Nature.

The DLP-printed hydrogel of Sil-MA exhibited suitable rheological and mechanical properties, such as remarkable viscoelastic characteristics and compressive and tensile strength [43]. The 30% Sil-MA hydrogel showed high compressive strength (910 kPa) and very good tensile strength. Therefore, it was possible to conduct a dog’s tracheal end-to-end anastomosis by sutures due to the mechanical properties of the Sil MA hydrogel. The excellent printability of Sil-MA was observed when several types of structures were printed with it, including a scaffold, a replica of the Eiffel Tower, a human ear, a brain, a trachea, a heart, a lung, and blood vessels (Figure 4). The biocompatibility of the Sil-MA hydrogel was excellent even at higher concentrations of Sil-MA when printed with NIH/3T3-embeded hydrogel using a DLP printer. An SGMA-based (ring-shaped) tracheal cartilage-like hydrogel-encapsulated chondrocyte was tested in vitro for 4 weeks; it demonstrated a suitable cellular environment for cell proliferation and cartilage development.

## 3. Applications of 3D DLP-Printable SF-Based Bio-Ink

DLP 3D bioprinting is suitable for various determined exterior and interior complex configurations of several complex organs, such as the lungs, cartilage, and bone. Recently, 3D DLP printing with SF-based bio-ink has been comprehensively employed in several biomedical applications (Table 3).

### 3.1. Bone Tissue Engineering

Bone is one of the most extensively transplanted tissues. Bone injuries may occur for various reasons, including trauma and medical diseases. Although bone has the unique property of self-repairment without scarring, bone replacement or additional surgery are usually needed if the bone defect is significant [70,71]. The advancement of 3D-printing technology allows for the creation of precise bone scaffolds and the encapsulation of various cells within the scaffolds to create bionic bone structures. DLP 3D printing has proven to be a good option for building these scaffolds. Dean et al. constructed a highly accurate bone scaffold via DLP 3D bioprinting [72]. This scaffold provided a suitable environment for cell proliferation and bone formation. For bone tissue engineering, the scaffold materials under consideration should offer matrix toughness while enabling extracellular matrix deposition. SF offers a high degree of toughness, mechanical strength, and biocompatibility. Therefore, SF-based materials have been widely applied in the fabrication of bone tissue scaffolds [34,73,74]. Recently, Rajput et al. developed an SF-based bio-ink for 3D DLP for a bone tissue scaffold using photocurable methacrylated SF (SF-MA) [66]. The DLP 3D-printed SF-MA hydrogels exhibited viscoelastic behavior while functioning as bone tissue and an excellent compressive modulus extending from ≈12 kPa to ≈96 kPa through rheological and mechanical characterizations. DLP-bioprinted SF-MA hydrogels proficiently enhanced the encapsulated pre-osteoblast growth with a satisfactory cell structure and cytoskeletal morphology. Osteogenesis was confirmed with an advanced degree of cell-mediated calcium deposition for a period of 14 days, indicating the potential of SF-based bio-inks in bone tissue engineering using DLP 3D-bioprinting technology.

### 3.2. Cartilage Tissue Regeneration

**Figure 5:**
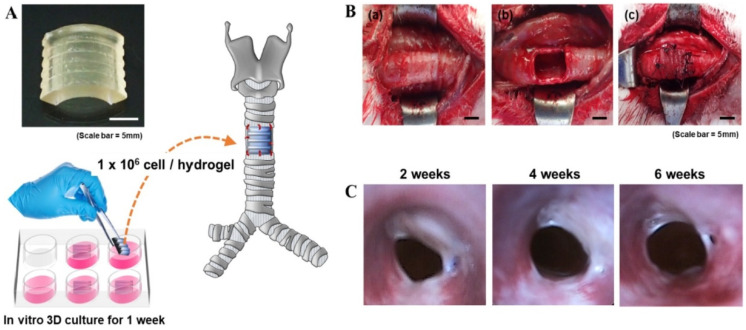
Schematic presentation of chondrocyte-encapsulated SF-GMA hydrogel implantation and endoscopic evaluation of rabbit trachea 6 weeks post-implantation. (**A**) Artificial trachea was fabricated using a DLP printer with chondrocyte-encapsulated SF-GMA and cultivated for 1 week in vitro. (**B**) (**a**,**b**) After surgery and elimination of the incised portion of the trachea, (c) fabricated trachea was transplanted. Scale bars present 5 mm. (**C**) Endoscopy was performed after 2, 4, and 6 weeks from trachea implantation. SF-GMA hydrogel exhibited the inner length progressively increased after implantation, and surrounding tissues developed in the operating area after 6 weeks of transplantation. Adapted from [75] with permission from Elsevier.

Cartilage, a type of connective tissue, is covered by a condensed extracellular matrix. Unlike bone tissue, cartilage cannot repair itself after injury. The cartilage deposition and repair processes depend on the chondrocyte’s action. The in vivo process of cartilage development—with chondrocytes in lacunae that control extracellular matrix (ECM) production, including glycosaminoglycan (GAG) synthesis—is similar to native cartilage [75,76]. Several surgical procedures, such as micro-fracture or engineered chondrocytes with cytokines and growth factors, have been applied in cartilage treatment [77,78]. However, there are still some drawbacks to treating significant cartilage defects with tissue-engineering technologies that must be overcome for efficient cartilage regeneration. In several studies, SF has enhanced cartilaginous extracellular matrix production due to its tunable morphologic features [79]. Previous studies reported that SF scaffolds seeded with human mesenchymal stem cells (hMSCs) could produce a zonal morphology identical to native cartilage tissue [79]. Furthermore, SF scaffolds loaded with insulin-like growth factor I (IGF-I) have been shown to promote chondrogenic differentiation in hMSCs [80]. Several other studies also demonstrated that blending SF with other biomaterials, including chitosan and gelatin, is a very promising method for cartilage regeneration [30,81]. In our previous study, we developed a modified SF with glycidyl methacrylate (GMA) (SGMA) and an SF bio-ink for DLP 3D printing-based cartilage regeneration [43]. The cartilage formation ability of human chondrocyte-laden SGMA hydrogels was evaluated in vitro. The chondrocyte-encapsulated-3D-printed hydrogel showed high chondrocyte viability and proliferation, leading to cartilage formation. From our results, we suggested that the DLP 3D-printed SF-GMA hydrogel containing chondrocyte may serve as a novel strategy for cartilage tissue engineering [43]. Recently, Hong et al. evaluated SGMA bio-ink-based 3D DLP printing for tracheal cartilage regeneration [82]. They also showed that the compressive properties of the SF-GMA were appropriate for cartilage regeneration both in vitro and in vivo. In previous studies, a 30% SF-GMA hydrogel exhibited high compressive strength (910 ± 127 kPa) after 3D DLP printing. It also showed a higher compressive modulus (125.8 ± 34 kPa) than gelatin-blended polycaprolactone (PCL) (75–94 kPa) or GelMA hydrogels (88 kPa) [43,83]. After in vitro chondrogenesis, the cell-encapsulated SGMA hydrogel was transplanted in a rabbit tracheal defect model. It was found that the SGMA-originated tracheal-shaped structure was firmly sutured with the adjacent tracheal tissue of the host. In addition, the sutured structure was steady for 6 weeks after implantation (Figure 5). This study also revealed that epithelium and neo cartilage-like tissue grew surrounding the implanted SGMA hydrogel after 6 weeks of implantation. This study suggested that SGMA hydrogels created with DLP 3D printing could be employed in tissue-engineering applications requiring strong physical properties, such as cartilage regeneration.

### 3.3. 4D Bioprinting System Using 3D DLP Printer Based on SF Bio-Ink for Tissue Engineering

**Figure 6:**
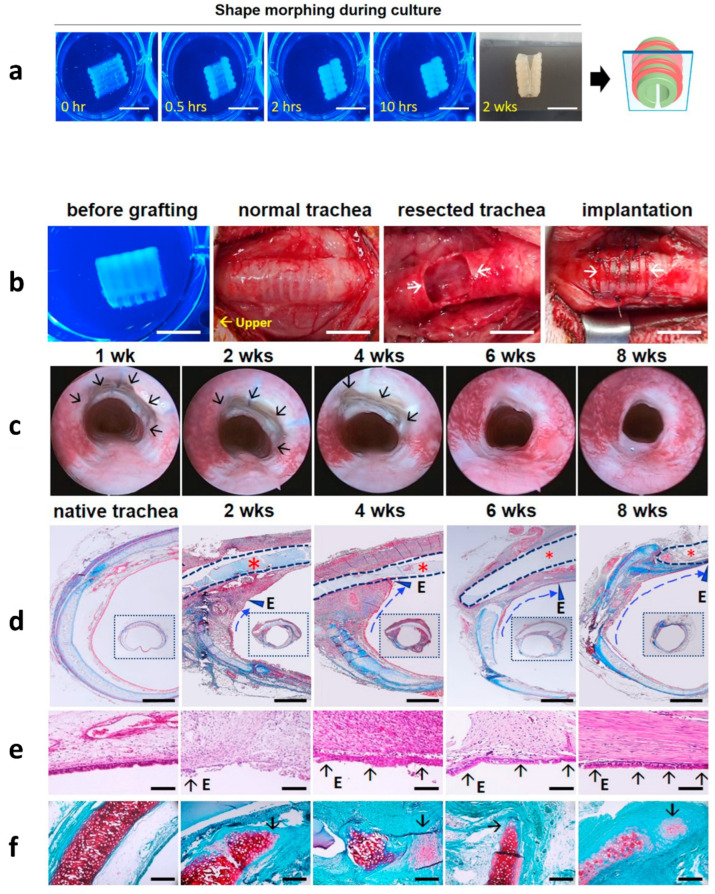
Implantation of the 4D-bioprinted trachea after in vitro culture. (**a**) Culture of 4D-bioprinted trachea in vitro. Shape morphing was achieved via in vitro culture: scale bars, 1 cm. (**b**) Transplantation of the fabricated trachea. The fabricated trachea was transplanted into an injured trachea rabbit model. Scale bars, 1 cm. (**c**) Bronchoscopic pictures of the intrinsic trachea and fabricated trachea at 1, 2, 4, 6 and 8 weeks post-implantation. Arrows indicate transplanted trachea. (**d**) Masson’s trichrome (MT) staining. The 4D-bioprinted trachea (marked as asterisks and dotted lines) was identified on the transplanted site: scale bars, 1 mm (1 cm for small boxes images). The area of the regenerated epithelium is marked as E and a dotted line with an arrowhead. (**e**) HE staining. Neo-respiratory epithelium (marked as E and short arrow) was identified at 2 weeks post-surgery. Scale bars, 100 μm. (**f**) Safranin O staining (immature cartilage is marked with a short arrow). This neo-cartilage had a greater cellular density and less dense sulfated glycosaminoglycan staining than normal tracheal cartilage: scale bars, 250 μm. Adapted from [39] with permission from Elsevier.

Four-dimensional (4D) bioprinting, a fusion of 3D printing and ‘Time,’ has emerged as a sensational new technology for tissue engineering applications. It can overcome some fundamental limitations of 3D bioprinting, including integration with the host tissue of the printed structure. It can also progress the functional responses of fabricated tissues, mimic the sophisticated dynamics of host tissues, and permit the employment of nominally aggressive surgical processes [84,85,86]. In 4D printing, exterior stimuli such as osmotic pressure [87], magnetic stimuli [88], temperature, electric current, and light [89,90] are the necessary stimuli for evolving the planar object created by a 3D printer to produce a predetermined construction over time. In this case, time refers to fabricated 3D-biocompatible scaffolds that retain changes over time during their fabrication process [91]. Shape-memorizing materials must be appropriate for usage in augmented 3D printers in order for 4D printing to modify their physicochemical characteristics in response to the necessary stimulus. However, the materials’ biocompatibility has not been considered in most 4D-printing studies. In addition, the external stimuli used for 4D printing are sometimes varied in tissue engineering applications [92,93]. Recently, a biocompatible and cell-friendly 4D-bioprinting system was reported using a photocurable SF (Sil-MA) hydrogel produced via a DLP 3D printer [47]. This 4D system created a preferred hydrogel form in a cell-friendly manner over a short period, resulting in good incorporation with respect to adjacent tissues at the implant site. After the shape was morphed, the authors fabricated hydrogels composed of two different layers (e.g., two different cell-laden bio-inks) (Figure 6).

The 4D-bioprinted (Sil-MA) trachea mimetic tissue with two cell types (human chondrocytes and MSCs) was successfully transplanted in an animal tracheal cartilage defect model. The newly formed respiratory epithelium and immature cartilage were revealed through histological analysis around the implanted hydrogel. In addition, the researchers fabricated complex structures, such as flowers, clams, and Dionaea muscipula. This study’s results suggested that this 4D system could be utilized to generate more complex tissues or organs in different target sites.

### 3.4. D DLP Printable Magnetic Bioreactor System Using SF Magnetic Bio-Ink for Muscle Tissue Regeneration

**Figure 7:**
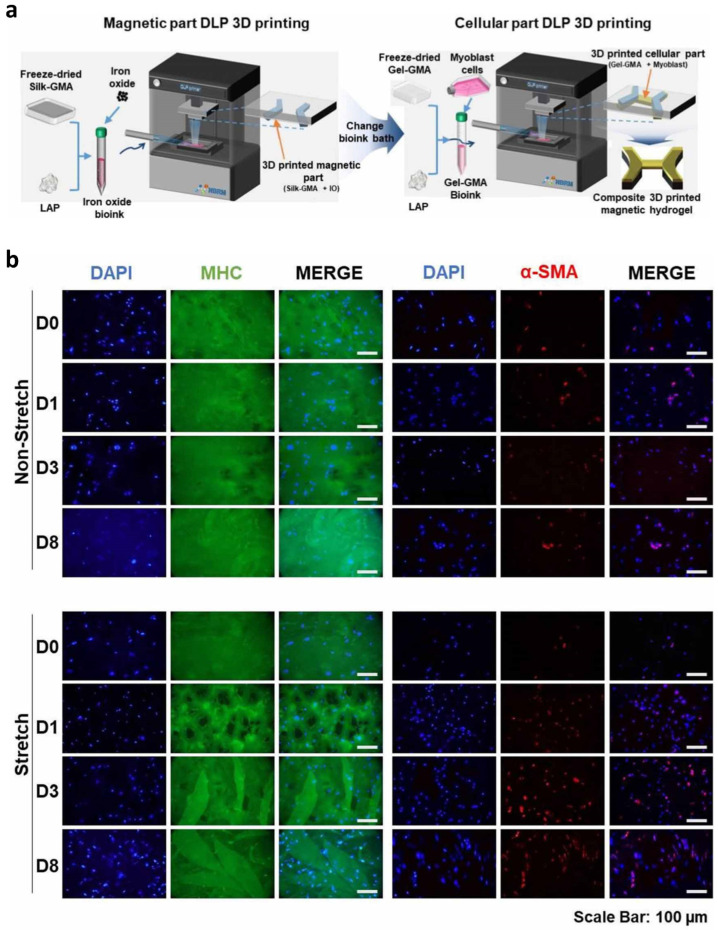
The fabrication process of magnetic hydrogel and the effect of magnetic bioreactor on magnetic hydrogel encapsulated myoblast cells. (**a**). Graphical presentation of the construction processes of the magnetic bioreactor in the 3D DLP printer. Silk-GMA + IO was used as a base layer bio-ink, while the top layers were printed with Gel-GMA + myoblast cells. Silk-GMA: methacrylated SF, Gel-GMA: methacrylated gelatin, GMA: glycidyl methacrylate, IO and O=Fe=O: iron oxide. (**b**). Fluorescence pictures exhibited myosin (MHC-green) and α-SMA (red) expressions for C2C12 cultured hydrogel in both static and stretched conditionThe alignment of the myotubes and α-SMA was similar to the direction of hydrogel. Adapted from [103] with the permission from IOP Publishing Ltd.

Bioreactor systems are frequently applied in modern tissue-engineering techniques to provide proper biophysical stimuli to tissue culture [94]. Several studies have evaluated cell differentiation using bioreactors that offer stimuli to cell-encapsulated material [95,96]. Numerous studies have revealed that mechanical stimuli prompted cellular alliance and muscle development in vitro and in vivo [97,98,99,100]. Therefore, several diverse approaches to mechanical stimulations are used in skeletal muscle engineering. Magnetic stimulation, for example, has been found to boost cellular activities and cell modulation so as to build cell clusters for complex tissue regeneration [101]. It has been demonstrated that a bioreactor system with both magnetic and mechanical stimuli towards the cell promoted muscle development and enhanced myotube diameter and length [102]. Recently, Ajiteru et al. developed a DLP 3D-printed magnetic bioreactor system using an SF magnetic bio-ink to differentiate myoblast cells [103]. Their study used a magnetic bioreactor system, and a magnetic hydrogel was fabricated using a DLP-printing technique. The fabricated hydrogel consisted of a magnetic part (iron oxide + SGMA) and a cellular part (encapsulated mouse myoblast cell (C2C12) in the gelatin methacrylate) (Figure 7). It was located in a magnetic bioreactor system to stimulate the cells in the hydrogel. It was shown that the magnetic bioreactor system enhanced the differentiation of mouse myoblast cells in the hydrogel with an increased diameter and length of the myotube in vitro (Figure 7). With the ability to stretch cells in three dimensions and print composite hydrogels using a one-stage layering process without any signs of cytotoxicity, this system can be used in the engineering of muscle tissue.

### 3.5. D DLP-Printable Fluorescent SF Bio-Ink for In Vitro and In Vivo Cell Tracking

Fluorescent hydrogels are frequently employed to observe non-invasive, real-time hydrogel deterioration and interactions with surrounding tissues [104,105]. In tissue regeneration, monitoring the hydrogel in vivo is crucial since its biodegradable nature is closely related to its applications. A fast degradation of the transplanted hydrogel may cause inadequate support for tissue growth, while sluggish degradation can obstruct neo-tissue growth [106]. Besides monitoring the hydrogel’s degradation, embedded cell tracing is another practical use of fluorescent polymers. Therefore, to confirm the in vivo concurrent visibility of a transplanted hydrogel, several studies were conducted by inserting quantum dots, nanoparticles, and fluorescent labels into polymer hydrogels [107,108,109]. However, several problems related to these fluorescent materials, including reduced stability, biodegradability, and biocompatibility, hinder their in vivo use as biomaterial trackers [110,111].

**Figure 8:**
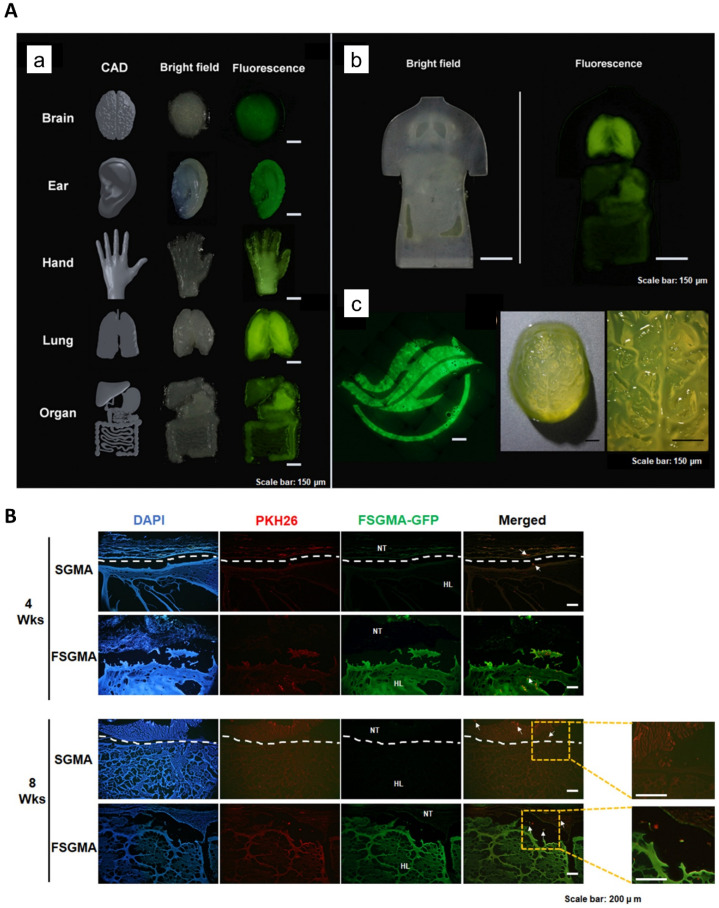
Printability test and FSGMA bio-ink for in vivo cell tracking. (**A**) FSGMA bio-ink’s printability. (**a**) The human brain, ear, hand, lung, and organs under bright and fluorescent fields. (**b**) Human trunk was printed with photocurable resin, and the internal organs were printed with FSGMA using DLP printer. Left side (bright field) and right side (fluorescence field). (**c**) Fluorescent image of the Hallym University logo and a mini human brain constructed using FSGMA. Scale bars present 150 μm. (**B**) Histological study of the FSGMA and SGMA hydrogel-encapsulated PKH26-tagged NIH3T3 cells. NT: native tissue and HL: hydrogel. White arrows indicate the presence of cell movement. Adapted from [114] with permission from Elsevier.

Fluorescent SF (FSF) is a natural polymer acquired through genetic engineering and has been presented in biomedical fields owing to its fluorescence characteristics. Previously, we applied FSF-tagged p53 (cancer marker) to detect cancer cells (HeLa) and proposed FSF as a bio imaging instrument for the real-time in vivo observation of stomach and esophageal damage [112]. Despite using FSF in several biomedical applications, the fabrication of a 3D form of fluorescent hydrogel has not yet been achieved due to the reduction in its fluorescent properties in gel structures [113]. Lee et al. recently reported a fluorescent SF-based 3D DLP printable bio-ink using FSF from genetically modified silkworms *(B. mori)* [114]. The glycidyl methacrylated fluorescent SF (FSGMA) was obtained through the covalent conjugation of FSF with GMA. The physical properties of the FSGMA hydrogel were similar to those of the SF (SGMA) hydrogel. It was possible to print several complex structures—including a human hand, ear, brain, lung, and interior organs—with this bio-ink by DLP 3D printer (Figure 8). The FSGMA hydrogel was also successfully used to track encapsulated cells in order to observe the in vitro and in vivo degradation of the scaffold. The authors suggested that 3D DLP-printable FSF bio-ink could play a substantial role in biomedical applications.

## 4. Conclusions

3D printing is a type of fast prototyping that uses a layer-by-layer method to construct items from CAD models. Recently, 3D-printing technology has become an attractive trend in biomedical fields. Despite the benefits of the DLP 3D-printing methods over other printing methods, such as their high resolution, fast printing times, and biocompatibility, they have not been commonly employed for bioprinting due to the small number of photosensitive and printable biomaterials available. SF is an FDA-approved natural polymer. It is well-known for its use in tissue-engineering fields owing to advantages such as its good mechanical properties, biocompatibility, and tunability. Recently, an SF-based bio-ink for 3D DLP printing was developed and produced from the methacrylate process [43]. It is biodegradable and biocompatible, has good mechanical properties, and shows remarkable printability in DLP-printing systems. In conjunction with other biomaterials, SF demonstrated its excellent potential to improve its physical properties for further applications. However, its clinical uses are still limited and require additional studies for clinical application and FDA-approved materials based on SF. Furthermore, several problems need to be solved; first, SF has been reported to demonstrate weak adhesion and low growth for a few cells (e.g., nerve cells) [67]. Thus, scientists recommend that SF should be combined with other materials, such as extracellular matrix or synthetic peptides, to improve its usefulness and application prospects. Second, in the printing process, UV radiation can cause cytotoxicity and gene mutation [115]. Although visible light is now applied in the printing process, additional studies on this topic may be required. Third, photo-initiators, including LAP and Irgacure 2959, can be cytotoxic [116,117]. LAP is a frequently used photo-initiator in 3D DLP printing. Although LAP is less cytotoxic than Irgacure 2959, the free radicals generated using LAP during bioprinting are potentially cytotoxic and mutagenic. The development of non-cytotoxic photo-initiators may be required for the future application of 3D DLP-printable silk-based bio-inks. This review shows that 3D DLP-printable SF-based bio-ink has various applications, including as bone tissue scaffolds, in cartilage regeneration, as an SF magnetic bio-ink, as an electroconductive biocomposite bio-ink, as fluorescent bio-ink, and as bio-ink for 4D-bioprinting systems for several biomedical applications. Despite significant challenges, 3D DLP-printable SF-based bio-ink has great potential for future DLP 3D bioprinting-based biomedical applications.

## Figures and Tables

**Table 1:** Comparison of 3D DLP bioprinting with conventional 3D-printing technology including extrusion-based and inkjet-based bioprinting [6,7,11,12].

Types	Materials	Advantages	Disadvantages	Methods of Crosslinking
Extrusion-based bioprinting	Thermoplastic polymer	1. Can employ high-viscosity bio-ink 2. Scalability 3. High cell concentration	1. Low cell viability (40–80%) 2. Slow (μm/s) printing speed 3. Lowest resolution 3. Nozzle clogging 4. Shear-thinning bio-ink	Chemical crosslinking
Inkjet-based bioprinting	Thermoplastic polymer	1. High resolution 2. High printing speed (mm/s 3. Affordable	1. Only well-suited to low-viscosity inks 2. Nozzle clogging 3. Cell damage	Enzymatic/sonication Chemical crosslinking
DLP bioprinting	Photosensitive polymer	1. Nozzle-free 2. Very fast printing speed (mm^3^/s) 3. High cell viability (85–95%) 4. Highest resolution	1. Bio-inks containing photo-initiator can cause cell damage 2. UV curing can damage DNA of cells	Photo-crosslinking

**Table 2:** Comparison of SF polymers with natural and synthetic polymers based on their major advantages and disadvantages [15,18,23,24,25,26,29,30,31].

Polymers	Advantages	Disadvantages
Synthetic (PCL, PEG, PLGA, PDMS)	Remarkable mechanical properties, convenient synthesis, mechanically and chemically stable, easy acquisition, easy processing, low cost, able to endure internal and external strains 3D for organ printing, excellent printability	Expensive synthesis process, immune rejection, poor biocompatibility, poor cellular adhesion, and production of toxic by-products during degradation
Natural (gelatin, alginate, fibrinogen, collagen, chitosan, HA	Low immunogenicity, good biocompatibility, good cell adhesion, supports cell migration and proliferation, fewer side effects, degradable, naturally abundant	Low mechanical properties, sometimes sever immunogenic, cause toxicity, inflammation, complex purification process, and poor printability
Silk Fibroin	Simple structural modification, controlled biodegradation, high cell viability, variety of crosslinking methods, and excellent mechanical strength	Low viscosity, individually very hard to print, and rheological properties need to be improved

Polycaprolactone (PCL), Polyethylene Glycol (PEG), Polylactic-co-glycolic Acid (PLGA), Polydimethylsiloxane (PDMS), and Hyaluronic acid (HA).

**Table 3:** Use of SF-based bio-ink for various biomedical applications via different 3D-printing methods [11,43,66,67,68,69].

Printing Methods	Year of Publication	Title	Contribution	Advantages	Disadvantages
DLP Bioprinting	2022	Light-based 3D bioprinting of bone tissue scaffolds with tunable mechanical properties and architecture from photocurable silk fibroin	Bone tissue engineering	Showed rheological and mechanical properties characteristic of human tissues. Good biocompatibility and cellular proliferation	Not significant
DLP Bioprinting	2018	Precisely printable and biocompatible silk fibroin bio-ink for digital light-processing 3D printing	Bone tissue engineering	Excellent biocompatibility, improved mechanical properties, good shape fidelity	Not significant
Extrusion-based 3D printing	2021	Crosslinker-free silk/decellularized extracellular matrix porous bio-ink for 3D bioprinting-based cartilage tissue engineering	Cartilage tissue engineering	Provides suitable chondrogenic differentiation cartilage repair environment	Poor shape fidelity; low-precision printing of objects, poor mechanical properties and degradion rate
Extrusion-based 3D printing	2021	3D printing of silk microparticle-reinforced polycaprolactone scaffolds; for tissue engineering applications	Bone tissue engineering	Effective mechanical properties, improved degradation rate, increased cell proliferation	Cytotoxicity observed with higher particle loading
Inkjet 3D printing	2020	Patterning the neuronal cells via inkjet printing of self-assembled peptides on silk scaffolds	Nerve tissue engineering	Allows for complex patterning of peptide nanofibers	Poor cell attachment
Inkjet 3D printing	2016	Inkjet printing of 3D silk fibroin cellular constructs using sacrificial alginate.	Vascular tissue engineering	Provides long-term metabolic activity	Poor biocompatibility

## Data Availability

Request upon corresponding author.
